# Comparative mitogenomic analyses of three scallops (Bivalvia: Pectinidae) reveal high level variation of genomic organization and a diversity of transfer RNA gene sets

**DOI:** 10.1186/1756-0500-2-69

**Published:** 2009-05-05

**Authors:** Xiangyun Wu, Xiaodong Xu, Ziniu Yu, Xiaoyu Kong

**Affiliations:** 1 Key Laboratory of Marine Bio-resource Sustainable Utilization, Laboratory of Applied Marine Biology; South China Sea Institute of Oceanology, Chinese Academy of Sciences, 164 West Xingang Road, Guangzhou 510301, PR China

## Abstract

**Background:**

It can be seen from the available mollusk mitogenomes that the family Pectinidae exhibits the most variation in genome organization. In this study, comparative mitogenomic analyses were performed for three scallops from the subfamily Chlamydinae (Pectinidae), with the goal of characterizing the degree of variability of mitogenome organization and other characteristics among species from the same subfamily and exploring their possible evolution route.

**Findings:**

The complete or nearly complete mtDNA sequences of scallop *Mimachlamys nobilis *(17 935 bp), *Mizuhopecten yessoensis *(20 964 bp) and *Chlamys farreri *(17 035 bp) were determined using long PCR amplification and primer walking sequencing strategy. Highly variable size difference of the three genomes resulted primarily from length and number variations of non-coding regions, and the major difference in gene content of the three scallop species are due to varying tRNA gene sets. Only 21, 16, and 17 tRNA genes were detected in the mitogenomes of *M. nobilis*, *M. yessoensis *and *C. farreri*, respectively. Remarkably, no *trnS *gene could be identified in any of the three scallops. A newly-detected *trnA*-like sequence within the mitogenome of *M. yessoensis *seems to exemplify the functional loss of a tRNA gene, and the duplication of *trnD *in *M. yessoensis *raises a fundamental question of whether the retention of the tRNA gene copy of 2-tRNAs is easier than that of 4-tRNAs. Analysis of putative evolutionary pathways of gene rearrangement indicates that transposition of neighboring gene blocks may play an important role in the evolution of mitogenomes in scallops. Parsimonious analysis of the genomic variations implies that the mitogenomes of *M. yessoensis *and *C. farreri *are likely to derive independently from a common ancestor that was closely related to *M. nobilis*.

**Conclusion:**

Comparative mitogenomic analyses among three species from the subfamily Chlamydinae show that the three genomes exhibit a high level of genomic variation and a diversity of tRNA gene sets, characterized by extensive translocation of genes. These features provide useful clues and information for evolutionary analysis of scallop mitogenomes.

## Findings

It can be seen from the available mollusk mitogenomes that the Pectinidae exhibits the most variation in genome organization. When we were initiating the current study, three complete or nearly complete mitogenomes, representing three subfamilies, were available from this family, i.e., *Argopecten irradians *(Aequipectini group, GenBank: EU023915), *Mizuhopecten yessoensis *(Chlamydinae, GenBank: AB271769) and *Placopecten magellanicus *(Palliolinae, GenBank: DQ088274). Obvious differences in mitogenome organization of three scallops were observed: 1) the sizes of three mitogenomes are distinct from each other, i.e. 16 221 bp for *A. irradians*, 20 414 bp for *M. yessoensis *and 32 115 bp for *P. magellanicus *[1]; 2) allegedly, the three mitogenomes have significantly different tRNA gene sets, with the numbers of 22, 32 and 9 for *A. irradians*, *P. magellanicus *and *M. yessoensis*, respectively; 3) the genomes show distinct gene arrangement patterns, namely unique rearrangements involving nearly every gene.

The degree of gene arrangements of mitogenomes from different species in the same subfamily, Chlamydinae, is one of our concerns. Therefore, in this study the complete mtDNA sequences of *Mimachlamys nobilis *and *M. yessoensis*, and nearly complete mtDNA sequence of *Chlamys farreri *are determined using long PCR amplification and primer walking sequencing strategy (see Additional file 1), and used for comparative analyses. Another reason for inclusion of *M. yessoensis *is that its first mitogenome data deposited in GenBank (AB271769) seems to bear significant omissions and mis-annotations of tRNA genes and protein-coding gene. Apparently, these mis-annotations need to be amended for further studies of gene order variation, evolution and phylogenetic analysis.

### Genome organization and nucleotide composition

The size of the mitogenome is 17 935 bp for *M. nobilis *(GenBank: FJ595958). Due to technical difficulties in sequencing, a small part (up to a couple of hundreds base pairs) of the mitogenome of *C. farreri *was not obtained and the nearly complete genome is 17 035 bp in length (GenBank: FJ595957). Genome assembly indicated that the unfinished section is the start part of the major non-coding region (MNR). The complete mtDNA sequence of *M. yessoensis *obtained in this study is 20 964 bp in length (GenBank: FJ595959), which is 550 bp longer than the nearly complete genome. Genome assembly indicated that the previously unfinished section is part of the MNR. Annotation to the mitogenome obtained in the current study and a re-annotation to AB271769 revealed the following findings: 1) the "absent" *cox2 *gene in previous annotation is actually present, corresponding to nucleotides 14 638–15 325, with "CTG" as initiation codon and "T" as termination codon; and 2) the genome has a total of 16 tRNA genes, instead of nine identified in that nearly complete genome. Additionally, 56 transitions were detected from a comparison of mitogenomes FJ595959 and AB271769.

**Figure 1:**
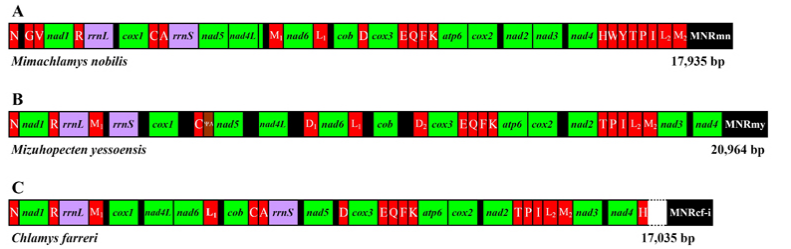
**Organization of the mitochondrial genome of *Mimachlamys nobilis *(A), *Mizuhopecten yessoensis *(B) and *Chlamys farreri *(C)**. Protein and rRNA coding genes are abbreviated as in the text, and transfer RNA genes are depicted by their corresponding one-letter amino acid code. Non-coding regions (>50 bp in length) are labeled by black box and the major non-coding region is designated as "MNRmn", "MNRmy" and "MNRcf-i", respectively.

The three mitogenomes contain 12 protein-coding genes (PCGs), lacking the *atp8 *gene as in most bivalves, two ribosomal RNA genes and varying numbers of tRNA gene set (see Additional file 2; Additional file 3; Additional file 4; Additional file 5; Figure 1). No obvious difference in gene length was observed among three scallops (Additional file 2); thus, what contributes to the differences in genome sizes is primarily the number and length of non-coding regions (NCRs). An interesting finding of this study is the existence of repeat units within the NCR of *M. yessoensis *mtDNA (see Additional file 6). The nucleotide compositions and usage frequencies of entire mitogenome for three scallops have a similar pattern (Additional file 2). The nucleotide compositions are all strongly skewed away from C in favor of G (the GC-skews are 0.312, 0.248 and 0.319 separately) and from A in favor of T (the AT-skews are -0.252, -0.197 and -0.240 respectively) (Additional file 2). The AT content of the three genomes are 59.6%, 55.3% and 57.9% in *M. nobilis*, *M. yessoensis*, and *C. farreri *respectively, lower than the average AT content of 16 available bivalves (61.6%), but comparable to that of other two pectinids (57% for *A. irradians*; 55.7% for *P. magellanicus*). Up to date, the five species within the Pectinidae represent the lowest average AT content (57.1%) compared to other molluscan lineages (66% for Gastropoda; 69.4 for Polyplacophora; 71.2% for Scaphopoda; 71.7% for Cephalopoda). The AT contents are slightly higher in *rrnL *(61.0%, 57.8% and 58% separately) and lower in *rrnS *(56.7%, 50.6% and 52.5%) than that of each full mitogenome.

### Protein-coding genes

Amino acid identity in proteins for pairs of three scallops ranged from as low as 54.3% in *nad2 *between *M. nobilis *and *C. farreri *to as high as 93.3% in *cox1 *between *M. yessoensis *and *C. farreri *(Table 1). *M. yessoensis *and *C. farreri *share a higher degree of amino acid similarity in all genes than do between other pairs. According to our data, it appears that the most conserved PCGs are *cox1*, *nad1*, and *nad3 *(identity > 80%), and the least conserved is *nad2 *(identity = 61.1%).

**Table 1:** Protein-coding gene assignments and identity of *Mimachlamys nobilis *(Mnob), *Mizuhopecten yessoensis *(Myes) and *Chlamys farreri *(Cfar)

**Protein**	**% identity**		
	
	**Mnob-Myes**	**Mnob-Cfar**	**Myes-Cfar**
*atp6*	65.8	65.8	75.3
*cob*	64.2	67	80.5
*cox1*	87	87.4	93.3
*cox2*	64.9	65.3	75.7
*cox3*	72.7	74.8	86.2
*nad1*	82.2	81.2	85.4
*nad2*	57.4	54.3	71.6
*nad3*	75.8	77.8	89.9
*nad4*	77.2	75.8	86
*nad4L*	72.1	71.2	85.6
*nad5*	71.9	68.6	77.3
*nad6*	64.8	67.9	75.9

In this study, most of PCGs in three mitogenomes use conventional initiation codons (20 for ATG, 10 for ATA and 1 for ATT), but two genes use the alternative ones (*M. nobilis*: *nad1*-TTG; *M. yessoensis*: *cox2*-CTG, *nad1*-GTG; *C. farreri*: *cox2*-GTG, *nad1*-GTG). Notably, one of the alternative initiation codons, GTG was also frequently used in two previously described scallop mitogenomes (5 for *A. irradians *and 2 for *P. magellanicus*). Only two genes use CTG as an initiation codon in the 45 reported molluscan mitogenomes (*M. yessoensis*: *cox2*; *Lottia digitalis*: *cox1*). Interestingly, six of 12 PCGs in *M. yessoensis *and *C. farreri *show a derived characteristic in their use of initiation codons when compared with those of *M. nobilis*, i.e. *atp6 *(ATA [*M. nobilis*]→ATG [*M. yessoensis *and *C. farreri*]), *cox1 *(ATA→ATG), *cob *(ATA→ATG), *nad1 *(TTG→GTG), *nad2 *(ATG→ATA) and *nad4 *(ATG→ATA). In general, the usage of initiation codons among three scallops is flexible, but not random.

Three genes stop with identical termination codon in all three scallops, i.e. *nad1 *(TAG), *nad4 *(TAG) and *nad4L *(TAA). Five genes (*atp6*, *cox2*, *cox3*, *cob *and *nad2*) share the same termination codon in the mitogenomes of *C. farreri *and *M. nobili *and three genes (*cox1*, *nad3 *and *nad5*) use the same termination codon in the genomes of *M. yessoensis *and *M. nobilis*. The *nad6 *in *M. yessoensis *and *C. farreri *display the derivative feature of termination codon usage when compared with that of *M. nobilis *(TAA [*M. nobilis*]→TAG [*M. yessoensis *and *C. farreri*]). The *cox2 *gene in both *M. yessoensis *and *C. farreri *contains a truncated termination codon, ending with a single thymine.

### Transfer RNA genes

Despite an extensive search with the tRNAscan-SE [2] and by eye inspection, only 21, 16, and 17 tRNA genes were detected in the mitogenomes of *M. nobilis*, *M. yessoensis *and *C. farreri*, respectively (Additional file 7; Additional file 8; Additional file 9). Particularly, no *trnS *gene could be identified in any of the three scallops, and four tRNAs (*trnG*, *trnV*, *trnW *and *trnY*) were absent in the mitogenome of both *M. yessoensis *and *C. farreri*. Nevertheless, we presume that the loss of *trnW *and *trnY *in the mitogenome of *C. farreri *may be an artifact, due to unfinished sequencing of the region downstream of *trnH*. The loss of *trnS *may present the ancestral state for all three scallops of the subfamily Chlamydinae. The loss of the tRNA cluster "GV" in the mitogenome of *M. yessoensis *and *C. farreri *may present the first step of tRNA gene loss from the common ancestor of these two species belonging to the same tribe (Chlamydini). Additional derived features of tRNA gene loss and translocations were observed separately for both *M. yessoensis *and *C. farreri*, e.g. the deletions of *trnH*, *trnW*, *trnY *and *trnA*, and the duplication of *trnD *in the mitogenome of *M. yessoensis*.

**Figure 2:**
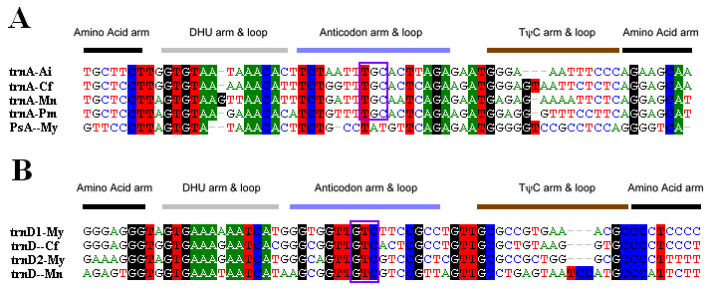
**Alignment of the *trnA *and pseudogene (PsA) in mitochondrial genomes of five scallops (A), and alignment of *trnD *in mitochondrial genomes of the three scallops (B)**. tRNA secondary structure is designed above the alignment, and the position of the anticodon is highlighted with rectangular frame. Ai: *Argopecten irradians *Cf: *Chlamys farreri *Mn: *Mimachlamys nobilis *My: *Mizuhopecten yessoensis *Pm: *Placopecten magellanicus*.

Another interesting finding of this study is the identification of a *trnA*-like sequence within the mitogenome of *M. yessoensis*. For confirmation of this finding, at least three individuals of *M. yessoensis *were used separately for amplification and sequencing of this fragment. This *trnA*-like structure is just located following the *trnC*, where the tRNA gene cluster "CA" has been observed in the mitogenome of both *M. nobilis *and *C. farreri*. Alignment of this *trnA*-like sequence of *M. yessoensis *with *trnA *gene sequences of other four available scallops (*A. irradians*, *P. magellanicus*, *M. nobilis *and *C. farreri*) shows that it shares similar nucleotide sequences in amino acid arm, DHU arm, and anticodon arm with those of *trnA *genes, with a distinct sequence in the anticodon loop (Figure 2A). We therefore define this *trnA*-like genomic region as a pseudo-tRNA gene (PsA). The existence of PsA may provide evidence of intermediate status of tRNA gene loss. Several mechanisms have been proposed for tRNA gene loss [3-5]. In this case, *trnA *gene loss seems fit the model that tRNA genes are gradually lost functionally, and then physically, over long evolutionary periods of time. Gene functional loss via mutation of the anticodon loop may play the most important role in this case.

In the mitogenomes of metazoan, almost all amino acids codons but leucine and serine are decoded by only one tRNA each [6]. The presence of two *trnM *genes was reported in the mitogenomes of tunicates [7]. However, it is a common phenomenon that mitogenomes of most bivalves contain two *trnM *genes. In this study, the *trnM*_2 _gene was found clustered with other tRNA genes, but the locations of *trnM*_1 _were variable over species. Another case of tRNA gene duplication is the *trnD *in *M. yessoensis*, which is found in single copy in the mitogenomes of both *M. nobilis *and *C. farreri*. Both *trnD*_1 _and *trnD*_2 _are considered true tRNA genes, based on sequence comparison (Figure 2B) as well as their putative secondary structures (Additional file 8). In order to probe into the mechanism of the retention of two copies of non-*trnL*/*trnS *tRNA genes, we examined the mitogenomes of all available mollusks for their tRNA gene usage, and found that a total of eight tRNA genes (*trnD*, *trnE*, *trnF*, *trnK*, *trnM*, *trnN*, *trnQ*, *trnV*) appear in at least two copies for each (see Additional file 10). Notably, all but *trnV *have anticodons corresponding to the 2-fold degenerate codons. This finding raises a fundamental question of whether the retention of the tRNA gene copy of 2-tRNAs is easier than that of 4-tRNAs (also see [6]). Perhaps there are differences in the gene loss and import mechanisms from the cytoplasm to the mitochondria of 4-tRNAs and 2-tRNAs. It is desirable to investigate the mechanisms of tRNA gene loss in further studies; more mitogenomes with variable tRNA gene sets should be included in future studies to draw a solid conclusion.

### Putative evolutionary pathway of gene rearrangements

**Figure 3:**
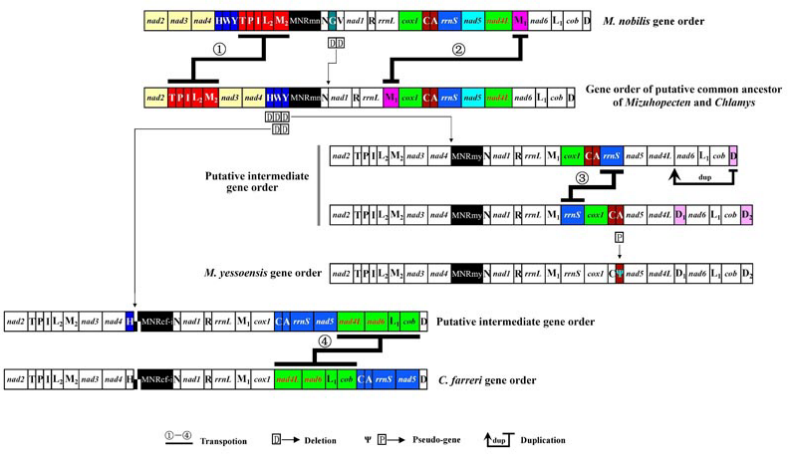
**The putative evolutionary pathway of mitochondrial genomes for three scallops**. The hypothetical pathway with the minimum number of gene duplication, deletion and transposition is illustrated. A putative common ancestor of *M. yessoensis *and *C. farreri *is shown as it can be most easily and parsimoniously explained. Putative intermediate gene order between "common ancestral gene order" and "*M. yessoensis*/*C. farreri *gene order" is also shown to explain the most parsimoniously possible evolutionary pathway of these two species. Symbols are explained under the figure and gene abbreviations are designated as in the text. Genes with identical order in the lineage are put in black/white boxes.

It is often difficult to trace the evolutionary pathway of metazoan mitogenomes due either to the generally low number of reorganization events (e.g., mammals) or drastic reorganizations in some animal lineages (e.g., nematodes, snails and brachiopods etc; see [8,9]), particularly in bivalves [e.g., [10,11]]. Phylogentic analyses for five scallops, using the concatenated amino acid sequences of 12 PCGs, resulted a high supported relationship which can be depicted as (*A. irradians*, (*P. magellanicus*, (*M. nobilis*, (*M. yessoensis*, *C. farreri*)))) (see Additional file 11). Based on the inferred phylogeny, a putative evolutionary pathway of gene rearrangement in the three species is assumed, and our preferred scenario of mitogenome evolution is further elaborated in Figure 3. In detail, there are at least three permutations between the mitogenomes of *M. nobilis *and that of a putative common ancestor of *M. yessoensis *and *C. farreri*: transposition of *trnM*_1_; transposition of the tRNA gene cluster "TPIL_2_M_2_", and loss of *trnG *and *trnV*. The tRNA gene cluster "HWY" is assumed to have been present in the putative common ancestor, based on the fact that *trnH *is just downstream of *nad4 *in *C. farreri*. Additionally, the absence of *trnW *and *trnY *may be artificial due to the incompletely sequenced downstream region. At least four independent events occurred in the mt gene rearrangement of *M. yessoensis *in the process of derivation from the putative common ancestor: deletion of the tRNA gene cluster "HWY", transposition of gene block "*cox1*-*trnC*-*trnA*" with *rrnS*, duplication of *trnD *and the generation of pseudogene PsA. Only one step is then necessary for the rearrangement of *C. farreri *from the common ancestor, i.e. the transposition of the large fragment "*trnC*-*trnA*-*rrnS*-*nad5*" with its neighbor block "*nad4L*-*nad6*-*trnL*_1_-*cob*".

It has been commonly recognized that variations in gene order are relatively rare in mitogenomes of most metazoan lineages [8]. Dowton et al. [12] estimated a probability of 1/2664 for a single event of gene translocation occurring independently in two mitogenomes (starting from the same gene order in both). However, this probability could be an underestimate according to yet unidentified constraints on modes of gene rearrangements, and it should be applied cautiously. Pectinid bivalves seem to represent another example, as five sequenced mitogenomes exhibit significant genomic rearrangements, suggesting that gene rearrangements occurred frequently among lineages in this family. On the other hand, high amino acid sequence identity between *M. yessoensis *and *C. farreri *indicate that these species may have diverged only recently. As illustrated in Figure 3, transposition of neighbor gene blocks (transposition 1, 3 and 4) may play an important role in the evolution of scallop mitogenomes. There is no doubt that mt genomic rearrangements are in most cases appropriate markers to resolve both ancient and recent divergence processes [6], but the result of this study implies that a careful estimation of the rearrangement pathway is especially required in analyzing the highly variable organizations of mitogenomes in the Pectinidae. However, the scallop mitogenomes with such diversity in their organization seem to be a good model to elucidate molecular evolutionary and phylogenetic issues of mitogenomes in future studies.

## Competing interests

The authors declare that they have no competing interests.

## Authors' contributions

XW designed the research, carried out most of the experiments, performed the data analyses and drafted the manuscript; XX and XK carried out part of the experiments and participated in data analyses; ZY initiated, led the research, supervised all laboratory work and finalized the manuscript. All authors have read and approved the final manuscript.

## Supplementary Material

Additional file 1 **Methods of PCR, sequencing and sequence analyses**. Detailed methods for molecular experiments and data analyses are described in this file. Click here for file

Additional file 2 **Basic information of mitochondrial genomes of three scallops**. This table presents the positions and nucleotide sequence lengths of mitochondrial genomes of three scallops, and initiation and termination codons for protein-coding genes as well as tRNA gene anticodons (starting from *trnN*). Click here for file

Additional file 3 **Organization of the mitochondrial genome of *Mimachlamys nobilis***. Protein and rRNA coding genes are abbreviated as in the text, and transfer RNA genes are depicted by their corresponding one-letter amino acid code. Non-coding regions (>50 bp in length) are labeled and the major non-coding region is designated as "MNRmn". Click here for file

Additional file 4 **Organization of the mitochondrial genome of *Mizuhopecten yessoensis***. Protein and rRNA coding genes are abbreviated as in the text, and transfer RNA genes are depicted by their corresponding one-letter amino acid code. Non-coding regions (>50 bp in length) are labeled and the major non-coding region is designated as "MNRmy". Click here for file

Additional file 5 **Organization of the mitochondrial genome of *Chlamys farreri***. Protein and rRNA coding genes are abbreviated as in the text, and transfer RNA genes are depicted by their corresponding one-letter amino acid code. Non-coding regions (>50 bp in length) are labeled and the incomplete sequenced major non-coding region is designated as "MNRcf-i". Click here for file

Additional file 6 **Analyses of non-coding regions and repeat units in mitogenomes of three scallops**. This section describes the unique character of non-coding regions and repeat units in mitogenomes of three scallops, including figure and references. Click here for file

Additional file 7 **Putative secondary structures for the 21 transfer RNA genes of the *Mimachlamys nobilis *mitogenome**. This figure show the putative secondary structures of tRNA generated by tRNAscan-SE 1.21. Click here for file

Additional file 8 **Putative secondary structures for the 16 transfer RNA genes of the *Mizuhopecten yessoensis *mitogenome**. This figure show the putative secondary structures of tRNA generated by tRNAscan-SE 1.21. Click here for file

Additional file 9 **Putative secondary structures for the 17 transfer RNA genes of the *Chlamys farreri *mitogenome**. This figure show the putative secondary structures of tRNA generated by tRNAscan-SE 1.21. Click here for file

Additional file 10 **List of tRNA usage frequency in 45 available mollusk mitogenomes**. This table shows the tRNA usage frequency in 45 available mollusk mitogenomes. Click here for file

Additional file 11 **Phylogentic analyses for five scallops, using the concatenated amino acid sequences of 12 PCGs**. Phylogenetic inferring were carried out using MEGA 4.1 for NJ (neighbor-joining), PAUP* 4b10 for MP (maximum parsimony) and PhyML for ML (maximum likelihood). Click here for file
