# Supplementary material for: Comparative mitogenomic analyses of three scallops (Bivalvia: Pectinidae) reveal high level variation of genomic organization and a diversity of transfer RNA gene sets
Source: BMC Res Notes. 2009 May 5;2:69. doi: 10.1186/1756-0500-2-69 (PMC2683862; doi:10.1186/1756-0500-2-69)
Supplement: Additional file 1 — Methods of PCR, sequencing and sequence analyses. Detailed methods for molecular experiments and data analyses are described in this file. [file 1756-0500-2-69-S1.doc]

**Methods**

***PCR and sequencing***

Specimen individuals of *M. yessoensis* and *C. farreri* were collected from Qingdao, Shandong province and that of *M. nobilis* from Dongshan, Fujian province, respectively. Total genomic DNA of each species was extracted from adductor muscle of single individual using sodium dodecylsulphate/proteinase K treatment, followed by spin-column purification (TIANamp Marine Animals DNA kit, Tiangen, Beijing). Primer pairs for amplification of mitogenome of *M. yessoensis* were designed based on the nearly complete mtDNA sequence available from GenBank (Table 1). Based on alignment and comparison of complete mitochondrial genome sequences of *A. irradians*, *P. magellanicus* and newly sequenced *M. yessoensis*, six primer pairs were designed for amplification of mtDNA large fragments of *M. nobilis* (Table 1). Similarly, a set of new primers were designed accordingly for amplification of *C. farreri* mitogenome based on mtDNA sequences of *M. yessoensis* and *M. nobilis*. PCR was performed in 25 μl reaction volume, containing 0.2 mM dNTP, 0.5 M of each primer, 1.0 U LATaq polymerase (TaKaRa), 2.0 mM MgCl_2_, 1× PCR buffer and 0.5 μl template DNA. PCR cycling conditions were 94℃ for 1 min; then 35 cycles of 94℃ for 20 sec, annealing temperature for 20 sec and 68℃ for 2-8 min, with a final extension at 72℃ for 10 min. PCR products were checked by electrophoresis on 1% agarose gel and purified using Qiagen PCR Purification kits (Qiagen, USA). Purified products were used as templates directly for cycle sequencing reactions. Species-specific primers for walking sequencing were designed and sequencing was performed for both strands of each fragment on an ABI 3730 DNA sequencer (ABI, USA).

***Sequence analyses***

During the processing of large fragments and those from walking sequencing, regular and manual examinations were used to ensure reliable overlapping and correct genome assembly. The mtDNA final consensus sequences were assembled using SeqMan (DNAstar, Madison, WI). Protein-coding and ribosomal RNA genes were firstly identified using BLAST searches at GenBank, and then by alignment with previously published mitogenomes of the two scallops and other closely related mollusks. Amino acid sequences of protein-coding genes were inferred with ORF Finder using invertebrate mitochondrial genetic code. Identification of tRNA genes was initially conducted with tRNAscan-SE [1] using mito/chloroplast genetic code and default search mode or setting the cove cutoff score to 1 when necessary, and the rest were identified by their potential secondary structures and anticodons. Gene map of the mitochondrial genomes were generated using CGView [2]. Nucleotide frequencies were calculated with DAMBE package [3]. The Relative Synonymous Codon Usage (RSCU) values were calculated with MEGA 4 [4]. Repeated regions were identified using Repeat Finder (<http://www.proweb.org/proweb/Tools/selfblast.html>).

**Table 1: Primer sequences for long PCR to amplify the mitochondrial in *Mizuhopecten yessoensis*, *Mimachlamys nobilis* and *Chlamys farreri***

| **Primer name** | **Sequence (5'-3')** | **Product size (bp)** |
| --- | --- | --- |
| ***M. yessoensis*** | | |
| X1For | AATTGAACGCTGCCTGAT (20,205-20,222) | 1197 |
| X1Rev | CACCCACAAAGAACCACA (420-437) |
| X2For | GTTCTTTGTGGGTGGTCT (424-441) | 6041 |
| X2Rev | TTTCCGCCATTTCTTTCT (6447-6464) |
| X3For | GGTGGTATTGATGCGTTGA (4512-4530) | 6033 |
| X3Rev | ATAGTTCTTCGCTTACCC (10536-10553) |
| X4For | GTTCTGTATGTGGCTTGTAT (9158-9177) | 6226 |
| X4Rev | TGGACCAGGTTCATTGCT (15366-15383) |
| X5For | CTATTCCAGGTCGGTTGA (15173-15190) | 4650 |
| X5Rev | CGAGAACGAAAGCAGAGT (19805-19822) |
| X6For | CGCCAAATGGATAGAAAG (19280-19297) | 3580 |
| X6Rev | GTCCGTTTAGGCTCGTAT (1878-1895) |
| ***M. nobilis*** | | |
| nad1F | TGGGGAAGTCTGTATGTG (644-661) | 1952 |
| 16SR | GATAACCAGAGCCAACAT (2578-2595) |
| 16SF | GAGTGAGAAAGACGAGA (2290-2306) | 1611 |
| cox1R | CATAGTTACCGCTGTGAA (3883-3900) |
| cox1F | TTTATTGAAGACGGGAGT (3319-3336) | 2317 |
| 12SR | GTCCACCTTCACCTGATACTT (5615-5635) |
| 12SF | GGATTTGGCGGCTCGTT (5360-5376) | 4236 |
| cytbR | AATGAAACTTTCCCCATC (9576-9593) |
| cytbF | TGTGGCTATTTGGATTTG (9299-9316) | 6175 |
| nad4R | CTATCCATTTGACGAAGA (15456-15473) |
| nad4F | CTATCCATTTGACGAAGA (15307-15324) | 3635 |
| nad1R | AAAGCCACCACTACCAAA (987-1006) |
| ***C. farreri*** | | |
| kND1F | AGCGATTTAGCCCTTTTA (124-141) | 2609 |
| k16SR | AAGGAAGATTACGCTGTTA (2714-2732) |
| k16SF | ATCCGCTTTGATGTTTGT (2488-2505) | 5670 |
| k12SR | CTGGAGGTTTCACATTTCTT (8437-8456) |
| k12SF | TAATCGACAGGGTCCGTAA (8345-8363) | 975 |
| kND5R | TTACCAGGGTAGATGAATG (9301-9319) |
| kND5F | TGTTGTTTAGAGGGATAGAG (9134-9153) | 7307 |
| kND4R | ACGAAGGCAGATAATAGAC (16422-16440) |
| kND4F | CTGTTTATCATCTCGTGCTT (16108-16127) | >1212 |
| kND1R | TACCAACATCCTCGCATA (267-284) |

**References**

1. Lowe TM, Eddy SR: **A program for improved detection of transfer RNA genes in genomic sequence.** *Nucl Acids Res* 1997, **25:**955-964.

2. Stothard P, Wishart DS: **Circular genome visualization and exploration using CGView.** *Bioinformatics* 2005, **21:**537-539.

3. Xia X, Xie Z: **DAMBE: software package for data analysis in molecular biology and evolution.** *J Hered* 2001, **92:**371-373.

4. Tamura K, Dudley J, Nei M, Kumar S: **MEGA4: Molecular evolutionary genetics analysis (MEGA) software version 4.0.** *Mol Biol Evol* 2007, **24:**1596-1599.
