# Supplementary material for: Comparative mitogenomic analyses of three scallops (Bivalvia: Pectinidae) reveal high level variation of genomic organization and a diversity of transfer RNA gene sets
Source: BMC Res Notes. 2009 May 5;2:69. doi: 10.1186/1756-0500-2-69 (PMC2683862; doi:10.1186/1756-0500-2-69)
Supplement: Additional file 2 — Basic information of mitochondrial genomes of three scallops. This table presents the positions and nucleotide sequence lengths of mitochondrial genomes of three scallops, and initiation and termination codons for protein-coding genes as well as tRNA gene anticodons (starting from trnN). [file 1756-0500-2-69-S2.doc]

**Table 1: Positions and nucleotide sequence lengths of mitochondrial genomes of *Mimachlamys nobilis* (Mnob), *Mizuhopecten yessoensis* (Myes) and *Chlamys farreri* (Cfar), and initiation and termination codons for protein-coding genes as well as tRNA gene anticodons (starting from *trnN***)

| **Genes** | **Positions and nt sequence lengths (bp)** | | | **Initiation and termination codons (Ini/Ter)** | | | **Anticodons** | | |
| --- | --- | --- | --- | --- | --- | --- | --- | --- | --- |
|  | **Mnob** | **Myes** | **Cfar** | **Mnob** | **Myes** | **Cfar** | **Mnob** | **Myes** | **Cfar** |
| *trnN* | 1-66 (66) | 1-66 (66) | 412-477 (66) |  |  |  | GTT | GTT | GTT |
| *trnG* | 191-257 (67) |  |  |  |  |  | TCC |  |  |
| *trnV* | 269-335 (67) |  |  |  |  |  | TAC |  |  |
| *nad1* | 341-1288 (948) | 70-1017 (948) | 481-1428 (945) | TTG/TAG | GTG/TAG | GTG/TAG |  |  |  |
| *trnR* | 1293-1358 (66) | 1022-1086 (65) | 1431-1495 (65) |  |  |  | TCG | TCG | TCG |
| *rrnL* | 1399-2773 (1375) | 1125-2548 (1424) | 1536-2966 (1431) |  |  |  |  |  |  |
| *cox1* | 2971-4596 (1626) | 4522-6174 (1653) | 3274-4917 (1644) | ATA/TAG | ATG/TAG | ATG/TAA |  |  |  |
| *trnC* | 4659-4724 (66) | 7276-7345 (70) | 7457-7526 (70) |  |  |  | GCA | GCA | GCA |
| *trnA* | 4740-4806 (67) |  | 7544-7610 (67) |  |  |  | TGC |  | TGC |
| *rrnS* | 4824-5769 (946) | 2891-3851 (961) | 7644-8601 (958) |  |  |  |  |  |  |
| *nad5* | 5827-7569 (1743) | 7439-9202 (1764) | 8761-10434 (1674) | ATG/TAA | ATA/TAA | ATG/TAG |  |  |  |
| *nad4L* | 7597-7920 (324) | 9406-9726 (321) | 5165-5482 (318) | ATG/TAG | ATG/TAG | ATG/TAG |  |  |  |
| *trnM* (M_1_) | 8140-8212 (73) | 2600-2671 (72) | 3188-3260 (73) |  |  |  | CAT | CAT | CAT |
| *nad6* | 8254-8748 (495) | 10024-10560 (537) | 5521-6015 (495) | ATG/TAA | ATA/TAA | ATG/TAG |  |  |  |
| *trnL* (L_1_) | 8752-8816 (65) | 10568-10631 (64) | 6022-6085 (64) |  |  |  | TAA | TAA | TAA |
| *cob* | 9003-10268 (1266) | 10812-11972 (1161) | 6266-7438 (1173) | ATA/TAG | ATG/TAA | ATG/TAG |  |  |  |
| *trnD* (D)*** | 10267-10335 (69) | 12548-12613 (66) | 10645-10710 (66) |  |  |  | GTC | GTC | GTC |
| *trnD* (D_1_) |  | 9957-10022 (66) |  |  |  |  |  | GTC |  |
| *cox3* | 10338-11201 (864) | 12617-13489 (873) | 10714-11565 (852) | ATG/TAA | ATG/TAG | ATG/TAA |  |  |  |
| *trnK* | 11243-11311 (69) | 13490-13559 (70) | 11606-11675 (70) |  |  |  | TTT | TTT | TTT |
| *trnF* | 11316-11378 (63) | 13568-13632 (65) | 11682-11745 (64) |  |  |  | GAA | GAA | GAA |
| *trnQ* | 11453-11523 (71) | 13707-13776 (70) | 11823-11893 (71) |  |  |  | TTG | TTG | TTG |
| *trnE* | 11531-11596 (66) | 13782-13847 (66) | 11900-11966 (67) |  |  |  | TTC | TTC | TTC |
| *atp6* | 11603-12478 (876) | 13863-14597 (735) | 11973-12761 (789) | ATA/TAG | ATG/TAA | ATG/TAG |  |  |  |
| *cox2* | 12487-13209 (723) | 14638-15325 (688) | 12785-13455 (671) | ATG/TAA | CTG/T | GTG/TA |  |  |  |
| *nad2* | 13295-14260 (966) | 15731-16711 (981) | 13919-14875 (957) | ATG/TAG | ATA/TAA | ATA/TAG |  |  |  |
| *nad3* | 14288-14620 (333) | 17096-17449 (354) | 15308-15610 (303) | ATG/TAA | ATG/TAA | ATA/TAG |  |  |  |
| *nad4* | 14749-15993 (1245) | 17569-18813 (1245) | 15727-16971 (1245) | ATG/TAG | ATG/TAG | ATG/TAG |  |  |  |
| *trnH* | 15995-16058 (64) |  | 16973-17035 (63) |  |  |  | GTG |  |  |
| *trnW* | 16066-16132 (67) |  |  |  |  |  | CCA |  |  |
| *trnY* | 16134-16199 (66) |  |  |  |  |  | GTA |  |  |
| *trnT* | 16219-16288 (70) | 16730-16779 (50) | 14894-14961 (68) |  |  |  | TGT | TGT | TGT |
| *trnP* | 16292-16358 (67) | 16808-16875 (68) | 14971-15038 (68) |  |  |  | TGG | TGG | TGG |
| *trnI* | 16362-16431 (70) | 16878-16947 (70) | 15045-15114 (70) |  |  |  | GAT | GAT | GAT |
| *trnL* (L_2_) | 16436-16503 (68) | 16950-17015 (66) | 15116-15182 (67) |  |  |  | TAG | TAG | TAG |
| *trnM* (M_2_) | 16507-16574 (68) | 17020-17085 (66) | 15186-15252 (67) |  |  |  | CAT | CAT | CAT |

* *trnD* was illustrated in Figure 1B as D_2_.
