# Supplementary figures and images for: Comparative mitogenomic analyses of three scallops (Bivalvia: Pectinidae) reveal high level variation of genomic organization and a diversity of transfer RNA gene sets
Source: BMC Res Notes. 2009 May 5;2:69. doi: 10.1186/1756-0500-2-69 (PMC2683862; doi:10.1186/1756-0500-2-69)

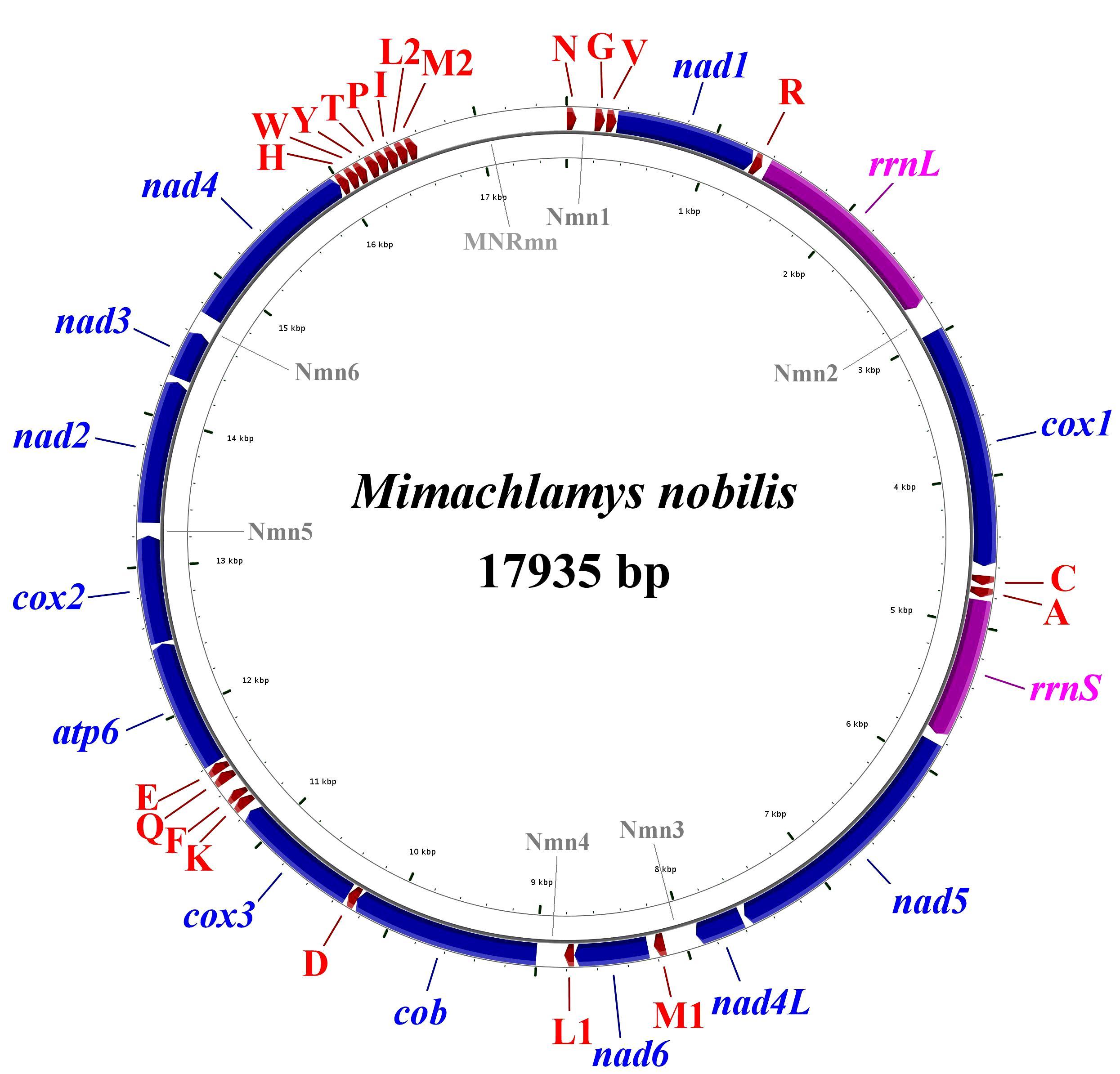

Supplement: Additional file 3 — Organization of the mitochondrial genome of Mimachlamys nobilis. Protein and rRNA coding genes are abbreviated as in the text, and transfer RNA genes are depicted by their corresponding one-letter amino acid code. Non-coding regions (>50 bp in length) are labeled and the major non-coding region is designated as "MNRmn". [file 1756-0500-2-69-S3.jpeg]

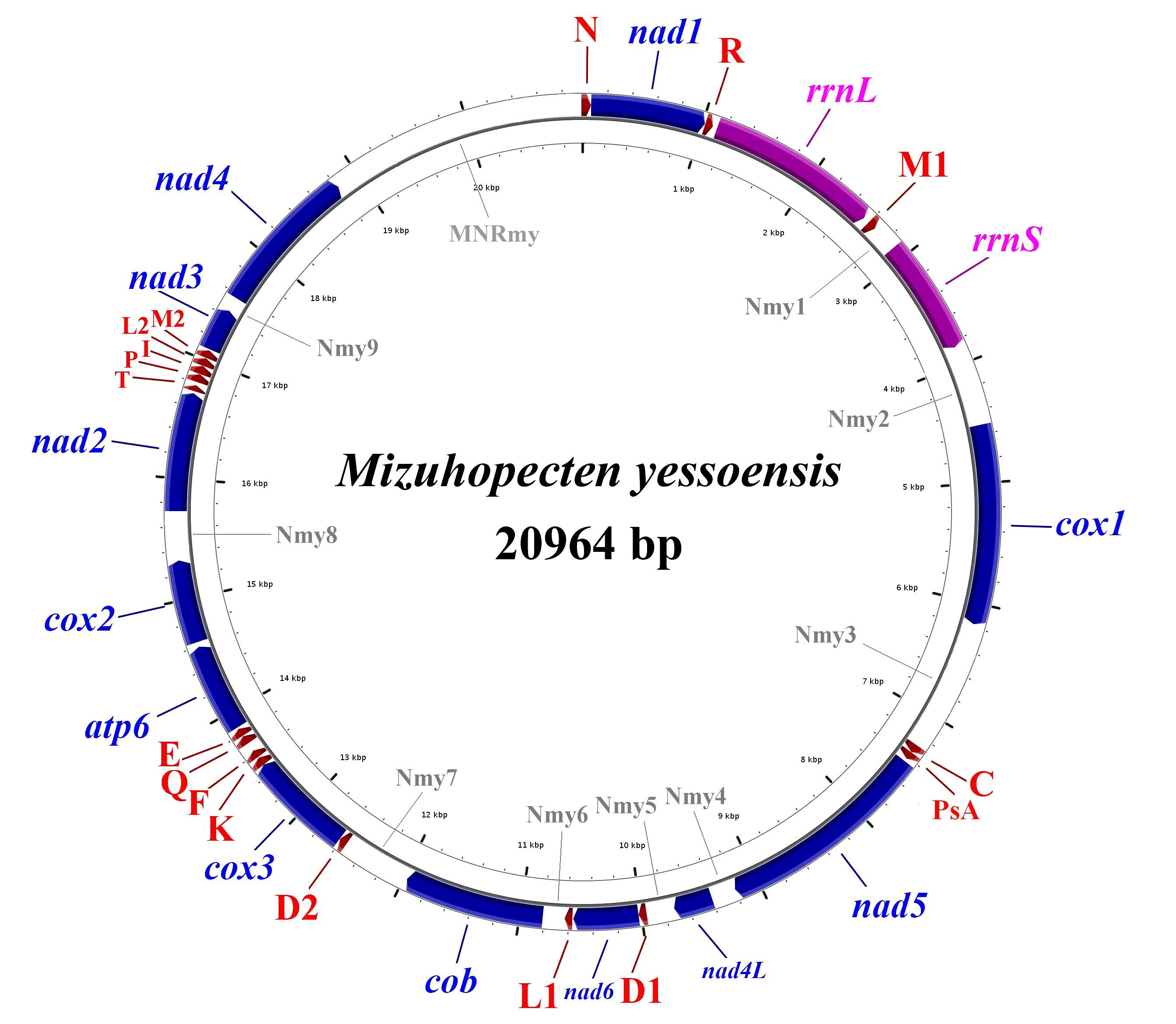

Supplement: Additional file 4 — Organization of the mitochondrial genome of Mizuhopecten yessoensis. Protein and rRNA coding genes are abbreviated as in the text, and transfer RNA genes are depicted by their corresponding one-letter amino acid code. Non-coding regions (>50 bp in length) are labeled and the major non-coding region is designated as "MNRmy". [file 1756-0500-2-69-S4.jpeg]

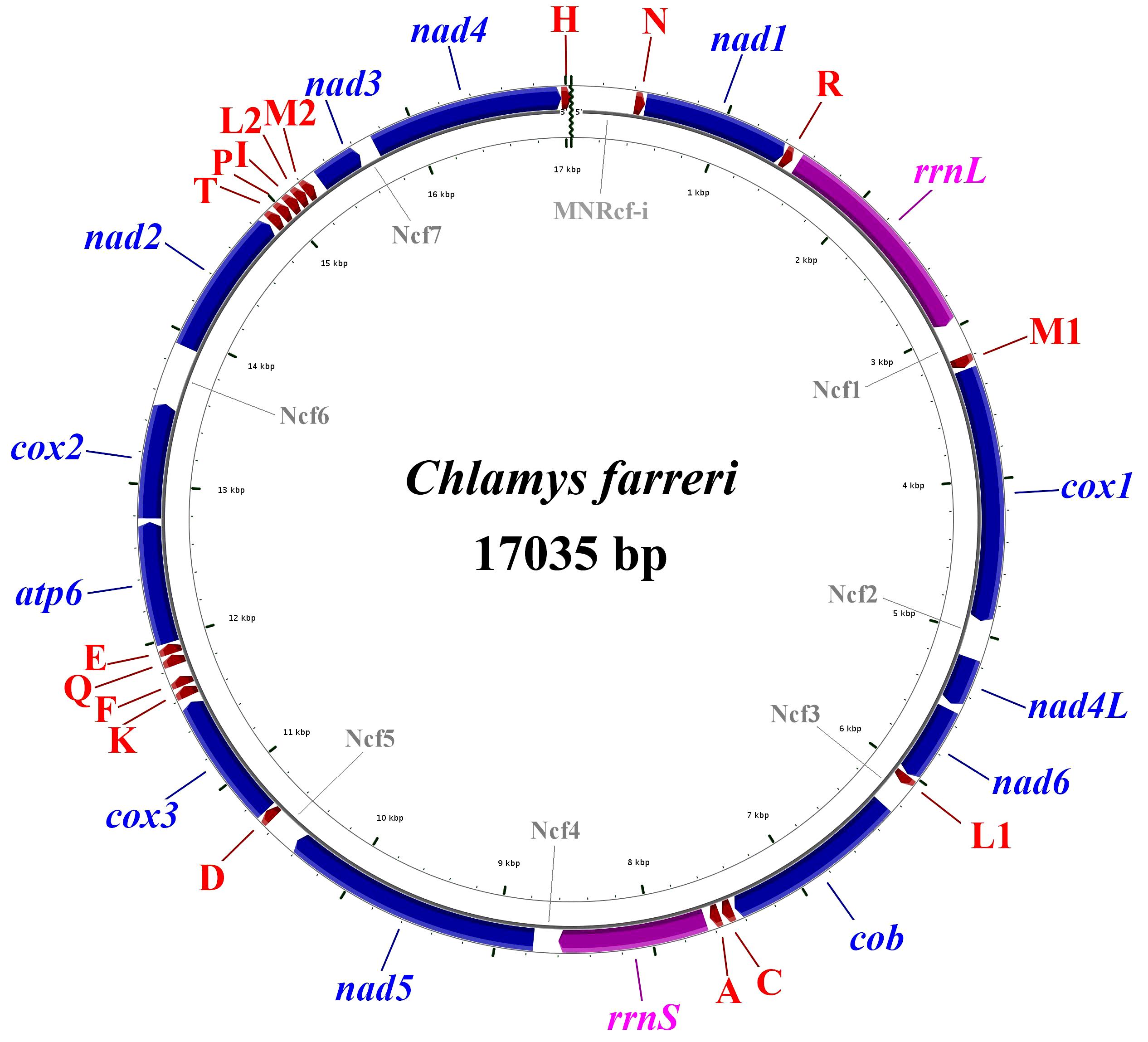

Supplement: Additional file 5 — Organization of the mitochondrial genome of Chlamys farreri. Protein and rRNA coding genes are abbreviated as in the text, and transfer RNA genes are depicted by their corresponding one-letter amino acid code. Non-coding regions (>50 bp in length) are labeled and the incomplete sequenced major non-coding region is designated as "MNRcf-i". [file 1756-0500-2-69-S5.jpeg]

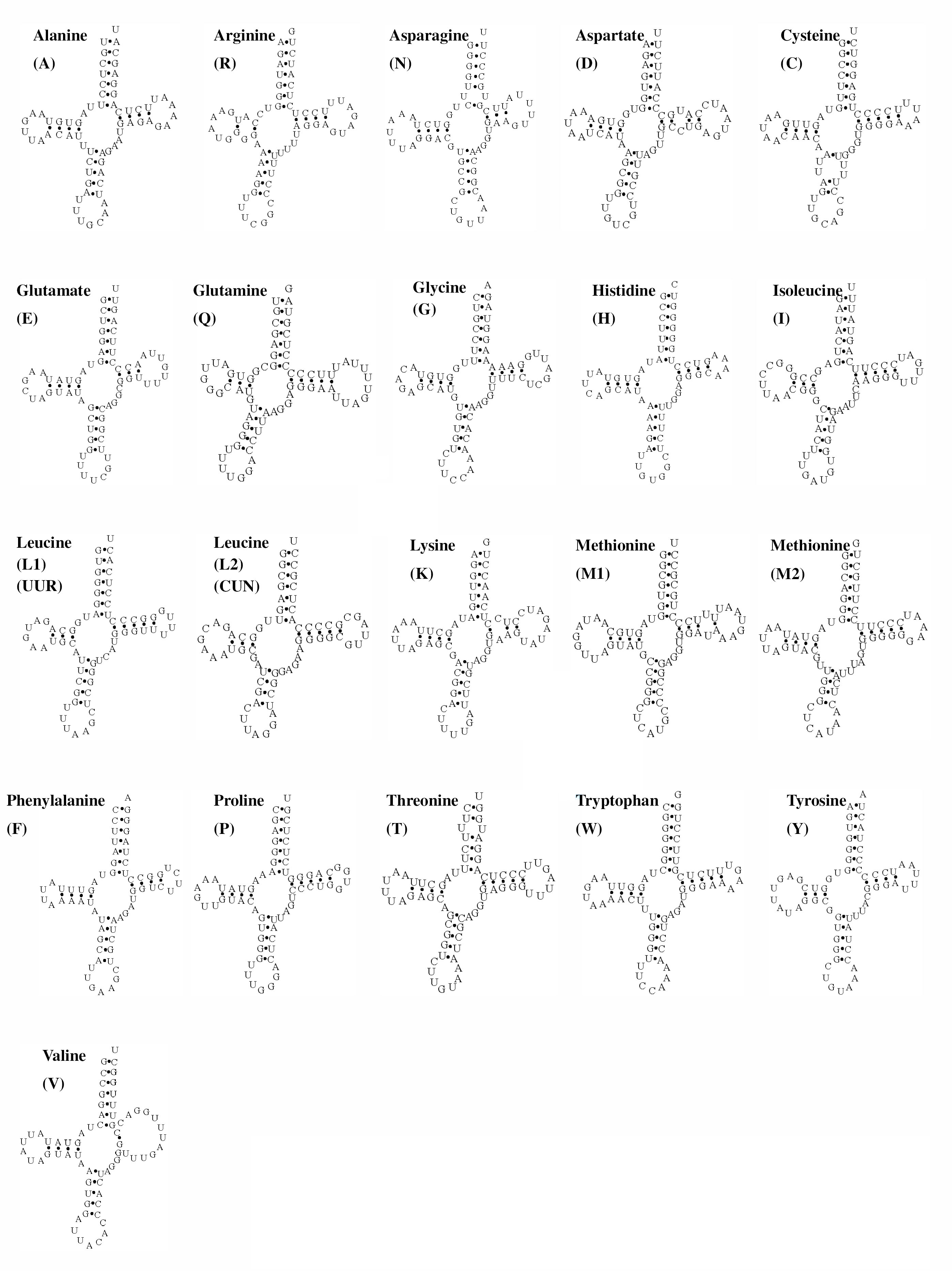

Supplement: Additional file 7 — Putative secondary structures for the 21 transfer RNA genes of the Mimachlamys nobilis mitogenome. This figure show the putative secondary structures of tRNA generated by tRNAscan-SE 1.21. [file 1756-0500-2-69-S7.jpeg]

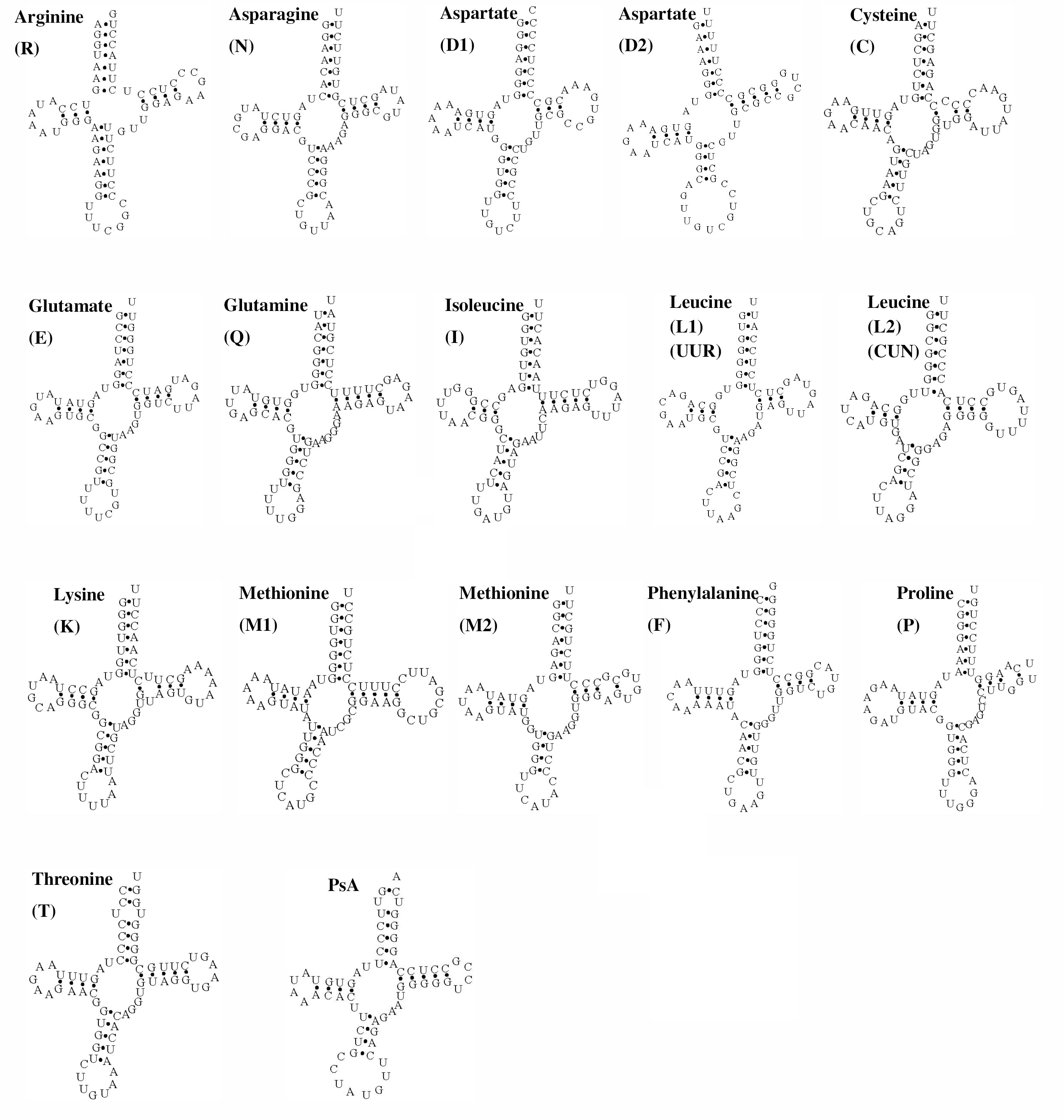

Supplement: Additional file 8 — Putative secondary structures for the 16 transfer RNA genes of the Mizuhopecten yessoensis mitogenome. This figure show the putative secondary structures of tRNA generated by tRNAscan-SE 1.21. [file 1756-0500-2-69-S8.jpeg]

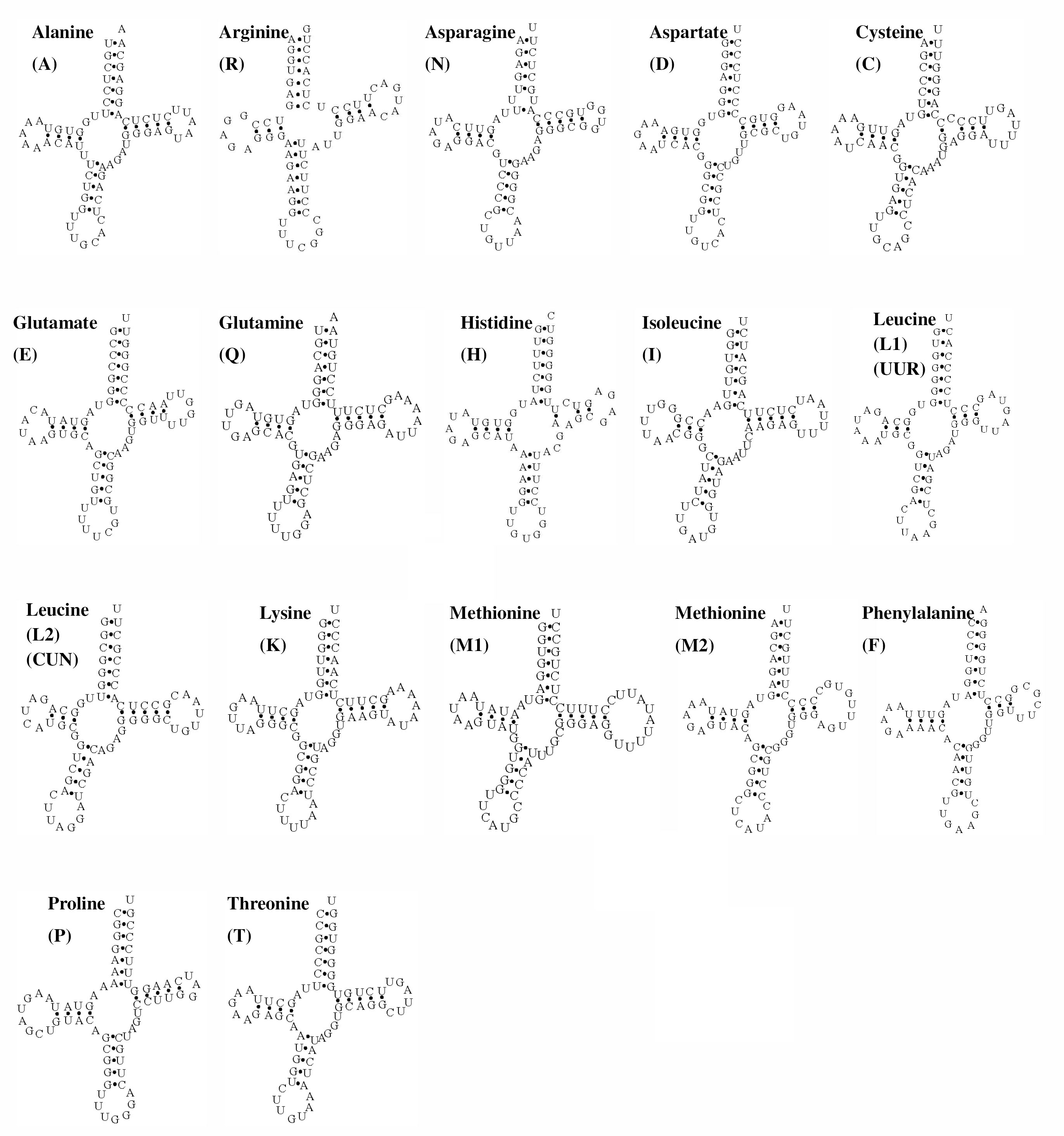

Supplement: Additional file 9 — Putative secondary structures for the 17 transfer RNA genes of the Chlamys farreri mitogenome. This figure show the putative secondary structures of tRNA generated by tRNAscan-SE 1.21. [file 1756-0500-2-69-S9.jpeg]

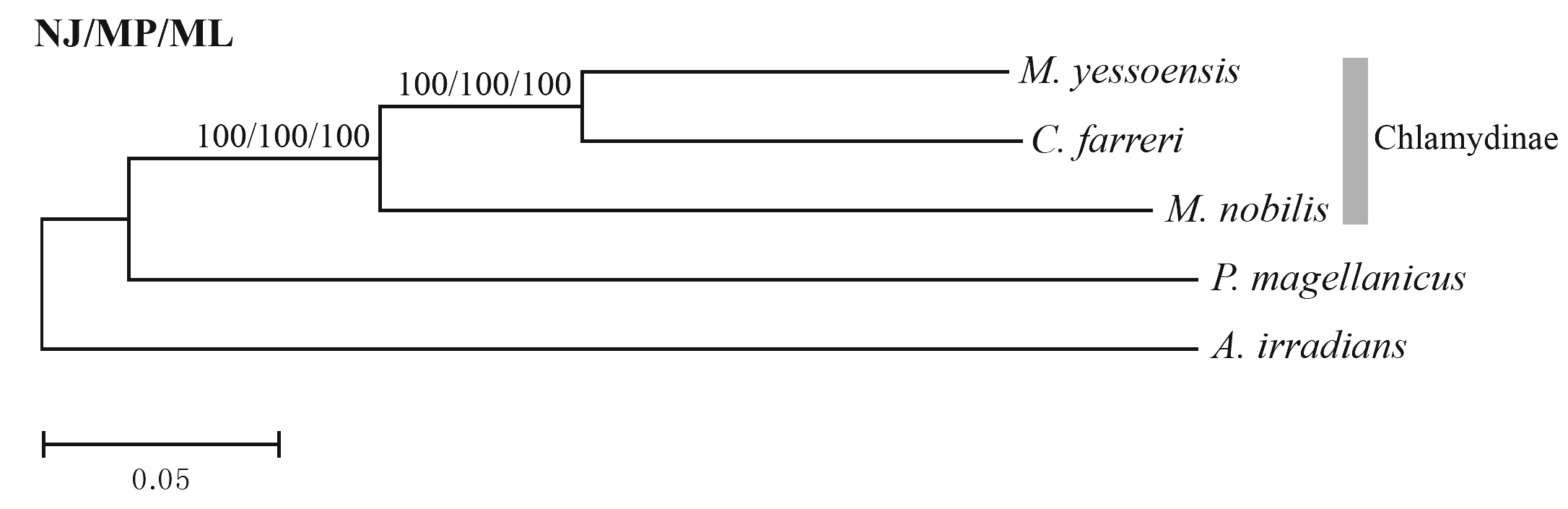

Supplement: Additional file 11 — Phylogentic analyses for five scallops, using the concatenated amino acid sequences of 12 PCGs. Phylogenetic inferring were carried out using MEGA 4.1 for NJ (neighbor-joining), PAUP* 4b10 for MP (maximum parsimony) and PhyML for ML (maximum likelihood). [file 1756-0500-2-69-S11.jpeg]
