# Supplementary material for: Comparative mitogenomic analyses of three scallops (Bivalvia: Pectinidae) reveal high level variation of genomic organization and a diversity of transfer RNA gene sets
Source: BMC Res Notes. 2009 May 5;2:69. doi: 10.1186/1756-0500-2-69 (PMC2683862; doi:10.1186/1756-0500-2-69)
Supplement: Additional file 6 — Analyses of non-coding regions and repeat units in mitogenomes of three scallops. This section describes the unique character of non-coding regions and repeat units in mitogenomes of three scallops, including figure and references. [file 1756-0500-2-69-S6.doc]

**Non-coding regions and repeat units**

The lengths of NCRs in the three newly sequenced scallop mitogenomes are highly variable; this variability results in different overall genome sizes. In *P. magellanicus*, more than 50% of the genome sequence are non-coding regions, consisting of dispersed, imperfectly repeated sequences that are associated with tRNAs or tRNA-like structures. These NCRs are responsible for the large genome size and abundance of tRNA genes [1]. In our case, we find the largest NCRs in *M. yessoensis* (Nmy1-9: 3 702 bp in total), in contrast to those of *M. nobilis* (Nmn1-6: 939 bp) and *C. farreri* (Ncf1-7: 1 521 bp). Though the MNR sequencing of *C. farreri* (MNRcf-i: 411 bp) is not completely finished, an obvious size difference is observed between *M. nobilis* (MNRmn: 1 360 bp) and *M. yessoensis* (MNRmy: 2 151 bp). In contrast to the comparatively compact mitogenomes of *M. nobilis* and *C. farreri*, the unusual size of NCRs (including MNR) in the mitogenome of *M. yessoensis* deserves further investigation.

Results showed that no repeat unit is found in the mitogenome of *M. nobilis* and only one repeat unit (“g”) was detected within the MNRcf-i in *C. farreri* (Figure 1A). However, a total of eight repeat units (“rep1-2”, “a”-“f”) were identified in the mitogenome of *M. yessoensis*, and six (“rep1”, “a”-“e”) in MNRmy (Figure 1A). The most interesting finding is the distribution of the repeat unit “rep1”, of which there are three copies: one embedded within the MNRmy, and other two dispersed, each nearly 6 kb and 7 kb away. Usually, tandem duplication mediated by replication error is the mechanism employed to form repeat sequences [2]. In this study, the ancestral locations of “rep1” and “rep2” are problematic when using the tandem duplication model as a mechanism to explain genome evolution in this case. A further examination with sequence alignment indicated that four NCRs from three scallops could be homologous: Nmn3 (downstream of *nad4L*, *M. nobilis*), Nmy4 (downstream of *nad5*, *M. yessoensis*), Nmy5 (downstream of *nad4L*, *M. yessoensis*) and Ncf5 (downstream of *nad5*, *C. farreri*) (Figure 1B). Considering that *M. nobilis* may have a close relationship with the ancestor of *M. yessoensis* (see the following section for detailed discussion), we presumed the ancestral location of “rep2” lies downstream of *nad4L* and it was copied via tandem duplication. The tandem duplication and random loss (TDRL) cannot be used here to explain the formation of “rep1”. The alternative model, non-tandem duplication mediated by illegitimate recombination via a mini-circle [3], may be more reasonable. The most parsimonious process of repeat region formation within the mitogenome of *M. yessoensis* is illustrated, in which “rep1” within the MNRmy is defined as the ancestral region and two non-tandem duplications arose separately (Figure 1C). Other repeat units existed in the MNRmy could easily be explained by TDRL model.


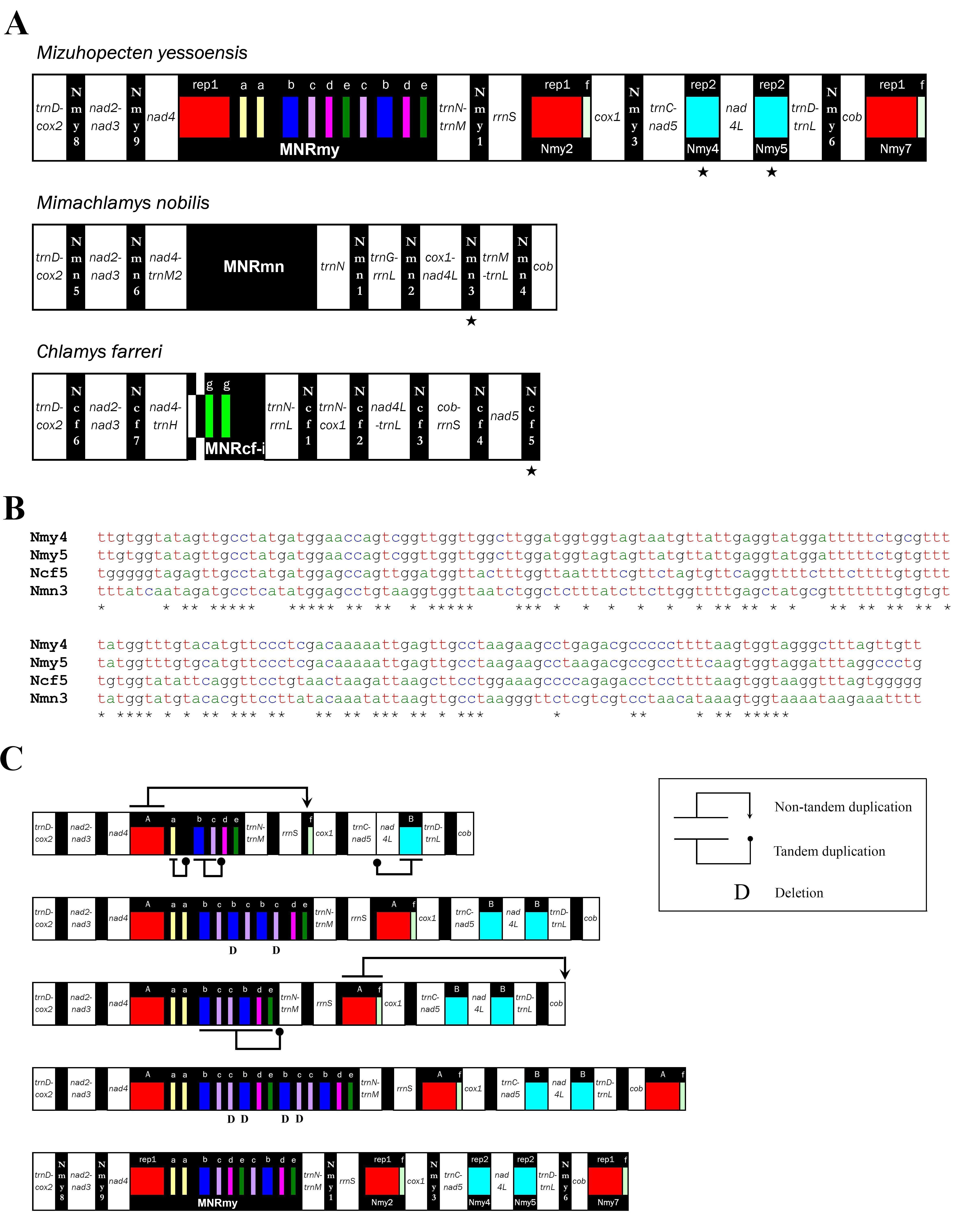


**Figure 1 - The locations, structure and comparative analyses of non-coding regions (NCRs) in the mitochondrial genomes of three scallops.**

(**A**) Structures of NCRs in three mitogenomes. Labels are the same with those in Figure 1-3 and the text. “rep1-2, a-g” indicates eight different repeat units. (**B**) Alignment of corresponding NCR sequences; puptative homolog are denoted with star symbol, (**C**) Putative evolutionary pathway of duplication in mt genomic NCRs of *Mizuhopecten yessoensis*.

**References**

1. Smith DR, Snyder M: **Complete mitochondrial DNA sequence of the scallop *Placopecten magellanicus*: Evidence of transposition leading to an uncharacteristically large mitochondrial genome.** *J Mol Evol* 2007, **65:**380-391.

2. Kurabayashi A, Sumida M, Yonekawa H, Glaw F, Vences M, Hasegawa M: **Phylogeny, reconbination, and mechanisms of stepwise mitochondrial genome reorganization in mantellid frogs from Madagascar.** *Mol Biol Evol* 2008, **25:**874-891.

3. Lunt DF, Hyman BC: **Animal mitochondrial DNA recombination.** *Nature* 1997, **387:**247.
