# Supplementary material for: Comparative mitogenomic analyses of three scallops (Bivalvia: Pectinidae) reveal high level variation of genomic organization and a diversity of transfer RNA gene sets
Source: BMC Res Notes. 2009 May 5;2:69. doi: 10.1186/1756-0500-2-69 (PMC2683862; doi:10.1186/1756-0500-2-69)
Supplement: Additional file 10 — List of tRNA usage frequency in 45 available mollusk mitogenomes. This table shows the tRNA usage frequency in 45 available mollusk mitogenomes. [file 1756-0500-2-69-S10.pdf]

List of tRNA usage frequency in 45 available mollusk mitogenomes

Taxon	tRNA usage frequency																				
	Sum	A	C	D	E	F	G	H	I	K	L	M	N	P	Q	R	S	T	V	W	Y
Bivalvia																					
Acanthocardia tuberculata	23	1	1	1	1	1	1	1	1	1	2	2	1	1	1	1	2	1	1	1	1
Argopecten irradians	22	1	1	1	1	1	1	1	1	1	2	1	1	1	1	1	2	1	1	1	1
Chlamys farreri	17	1	1	1	1	1	0	1	1	1	2	2	1	1	1	1	0	1	0	0	0
Crassostrea gigas	24	1	1	1	1	1	1	1	1	1	2	2	1	1	2	1	2	1	1	1	1
Crassostrea hongkongensis	21	1	0	1	1	1	1	1	1	1	2	2	0	1	1	1	2	1	1	1	1
Crassostrea virginica	23	1	1	1	1	1	1	1	1	1	2	2	1	1	1	1	2	1	1	1	1
Hiatella arctica	23	1	1	1	1	1	1	1	1	1	2	2	1	1	1	1	2	1	1	1	1
Lampsilis ornata	21	1	1	1	1	1	1	1	1	1	2	1	1	1	1	1	1	1	1	1	1
Mimachlamys nobilis	21	1	1	1	1	1	1	1	1	1	2	2	1	1	1	1	0	1	1	1	1
Mizuhopecten yessoensis	16	0	1	2	1	1	0	0	1	1	2	2	1	1	1	1	0	1	0	0	0
Mytilus edulis	23	1	1	1	1	1	1	1	1	1	2	2	1	1	1	1	2	1	1	1	1
Mytilus galloprovincialis	23	1	1	1	1	1	1	1	1	1	2	2	1	1	1	1	2	1	1	1	1
Mytilus trossulus	24	1	1	1	1	1	1	1	1	1	2	2	1	1	2	1	2	1	1	1	1
Placopecten magellanicus	34	1	1	1	2	4	1	1	1	2	2	9	2	0	1	0	3	1	1	1	1
Sinonovacula constricta	19	1	1	1	1	1	1	1	1	1	2	1	1	1	0	1	1	1	1	0	1
Venerupis philippinarum	23	1	1	1	1	1	1	1	1	1	2	2	1	1	1	1	1	1	2	1	1
Gastropoda																					
Albinaria coerulea	22	1	1	1	1	1	1	1	1	1	2	1	1	1	1	1	2	1	1	1	1
Aplysia californica	22	1	1	1	1	1	1	1	1	1	2	1	1	1	1	1	2	1	1	1	1
Biomphalaria glabrata	22	1	1	1	1	1	1	1	1	1	2	1	1	1	1	1	2	1	1	1	1
Biomphalaria tenagophila	22	1	1	1	1	1	1	1	1	1	2	1	1	1	1	1	2	1	1	1	1
Cepaea nemoralis	22	1	1	1	1	1	1	1	1	1	2	1	1	1	1	1	2	1	1	1	1
Conus textile	22	1	1	1	1	1	1	1	1	1	2	1	1	1	1	1	2	1	1	1	1
Elysia chlorotica	21	1	1	1	1	1	1	1	1	1	2	1	1	1	1	1	1	1	1	1	1
Haliotis rubra	22	1	1	1	1	1	1	1	1	1	2	1	1	1	1	1	2	1	1	1	1
Ilyanassa obsoleta	22	1	1	1	1	1	1	1	1	1	2	1	1	1	1	1	2	1	1	1	1
Lophiotoma cerithiformis	22	1	1	1	1	1	1	1	1	1	2	1	1	1	1	1	2	1	1	1	1
Lottia digitalis	20	1	1	1	1	1	1	1	1	0	2	1	1	1	1	1	1	1	1	1	1
Pupa strigosa	22	1	1	1	1	1	1	1	1	1	2	1	1	1	1	1	2	1	1	1	1
Roboastra europaea	22	1	1	1	1	1	1	1	1	1	2	1	1	1	1	1	2	1	1	1	1
Thais clavigera	22	1	1	1	1	1	1	1	1	1	2	1	1	1	1	1	2	1	1	1	1
Cephalopoda																					
Dosidicus gigas	23	1	1	2	1	1	1	1	1	1	2	1	1	1	1	1	2	1	1	1	1
Loligo bleekeri	22	1	1	1	1	1	1	1	1	1	2	1	1	1	1	1	2	1	1	1	1
Nautilus macromphalus	21	1	1	1	1	1	1	1	1	0	2	1	1	1	1	1	2	1	1	1	1
Octopus ocellatus	22	1	1	1	1	1	1	1	1	1	2	1	1	1	1	1	2	1	1	1	1
Octopus vulgaris	22	1	1	1	1	1	1	1	1	1	2	1	1	1	1	1	2	1	1	1	1
Sepia esculenta	22	1	1	1	1	1	1	1	1	1	2	1	1	1	1	1	2	1	1	1	1
Sepia officinalis	22	1	1	1	1	1	1	1	1	1	2	1	1	1	1	1	2	1	1	1	1
Sepioteuthis lessoniana	22	1	1	1	1	1	1	1	1	1	2	1	1	1	1	1	2	1	1	1	1
Sthenoteuthis oualaniensis	23	1	1	2	1	1	1	1	1	1	2	1	1	1	1	1	2	1	1	1	1
Todarodes pacificus	22	1	1	2	1	1	1	1	1	1	1	1	1	1	1	1	2	1	1	1	1
Vampyroteuthis infernalis	22	1	1	1	1	1	1	1	1	1	2	1	1	1	1	1	2	1	1	1	1
Watasenia scintillans	23	1	1	2	1	1	1	1	1	1	2	1	1	1	1	1	2	1	1	1	1
Polyplacophora																					
Katharina tunicata	22	1	1	1	1	1	1	1	1	1	2	1	1	1	1	1	2	1	1	1	1
Scaphopoda																					
Graptacme eborea	22	1	1	1	1	1	1	1	1	1	2	1	1	1	1	1	2	1	1	1	1
Siphonodentalium lobatum	22	1	1	1	1	1	1	1	1	1	2	1	1	1	1	1	2	1	1	1	1
